# Sex-disaggregated data matters: tracking the impact of COVID-19 on the health of women and men

**DOI:** 10.1007/s40888-021-00254-4

**Published:** 2022-01-20

**Authors:** Sarah Hawkes, Athena Pantazis, Anna Purdie, Abhishek Gautam, Sylvia Kiwuwa-Muyingo, Kent Buse, Sonja Tanaka, Kakoli Borkotoky, Sneha Sharma, Ravi Verma

**Affiliations:** 1 Institute for Global Health, UCL, and Global Health 50/50, 30 Guilford Street, London, WC1N 1EH UK; 2 Independent Consultant To Global Health 50/50, Maseru, Lesotho; 3 International Center for Research On Women, C-59, South Extension Part -2, New Delhi , 110049 India; 4 African Population and Health Research Center, APHRC Campus, Manga Close, Off Kirawa Road, P.O. Box 10787-00100, Nairobi, Kenya; 5 Healthier Societies Programme, The George Institute for Global Health and Global Health 50/50, University of New South Wales, Global Health 50/50, Paris, France; 6 Global Health 50/50, 16 A Rue Petit, Paris, France

**Keywords:** COVID-19, Sex-disaggregated data, Gender inequality, Health inequity, I14, I15, I18

## Abstract

Sex and gender matter to health outcomes, but despite repeated commitments to sex-disaggregate data in health policies and programmes, a persistent and substantial absence of such data remains especially in lower-income countries. This represents a missed opportunity for monitoring and identifying gender-responsive, evidence-informed solutions to address a key driver of the pandemic. In this paper we review the availability of national sex-disaggregated surveillance data on COVID-19 and examine trends on the testing-to-outcome pathway. We further analyse the availability of data according to the economic status of the country and investigate the determinants of sex differences, including the national gender inequality status (according to a global index) in each country. Results are drawn from 18 months of global data collection from over 200 countries. We find differences in COVID-19 prevention behaviours and illness outcomes by sex, with lower uptake of vaccination and testing plus an elevated risk of severe disease and death among men. Supporting and maintaining the collection, collation, interpretation and presentation of sex-disaggregated data requires commitment and resources at subnational, national and global levels, but provides an opportunity for identifying and taking gender-responsive action on health inequities. As a first step the global health community should recognise, value and support the importance of sex-disaggregated data for identifying and tackling an inequitable pandemic.

## Introduction

COVID-19 has served to both illustrate and, in some cases, exacerbate existing inequities in health and wellbeing. Exposure to the virus itself, as well as access to and provision of health services (both preventative and curative), have highlighted the social, occupational, structural, commercial and political drivers of the pandemic and its impact across communities and countries. Intersecting across all these drivers, sex and gender play important, and frequently overlooked, roles in determining the differential health impacts of COVID-19 on people and populations.

In this paper we refer to ‘sex’ as a binary categorisation of biological sex—i.e. a male or female person, with attendant differences in chromosomal, immunological, hormonal characteristics (Klein & Flanagan, 2016; Mauvais-Jardin et al., 2020). We use the term ‘gender’ to indicate socially and structurally determined inequalities that determine power, position and privilege associated with the identity of being a man, woman, transgender or non-binary person in any society, and reflect on how this determines health and wellbeing for all people (Hawkes & Buse, 2013; Heise et al., 2019).

Regular analysis of sex- disaggregated data reported by national (and occasionally sub-national) surveillance systems can shed light on areas for gender-responsive policy and programme action. For example, sex- disaggregated data, particularly if also disaggregated by age groups, can identify whether risks of poor health outcomes are higher in men or women in certain age groups (e.g. the elderly). Disaggregated data can also be used to monitor whether interventions/services are reaching all sections of the population equally – for example keeping track of whether some groups in the population are less likely to be reached by vaccine programmes. The United Nations General Assembly considered disaggregated data as essential for the “design, implementation and evaluation of effective policies in response to the COVID-19 pandemic” (United Nations General Assembly, 2020). Moreover, disaggregated data contributes to human rights-based accountability systems that monitor inequities across populations, and furnishes evidence for remedial actions to address and reduce inequities (Williams & Hunt, 2017).

Despite repeated commitments, including a Sustainable Development Goal (SDG) target (17.18) and United Nations guidance (UN General Statistical Commission, 2021) on disaggregated data in health to constitute a global norm, previous analyses have found persistent and substantial absences of sex-disaggregated data in both peer-reviewed publications (Sugimoto et al., 2019), programme data (Global Health 50/50, 2021), and vital registration system data (Mikkelsen et al., 2015). Nationally reported COVID-19 data is no exception: the data is limited in its coverage and consistency and degrees of disaggregation.

Apart from sex and age, national data are rarely disaggregated along other axes of inequality such as ethnicity, geographical location, occupation, or class (Agarwal, 2021a). When more intersectional data are (all too rarely) made available, clear patterns of health inequity can be found. Surveillance data from the United Kingdom have shown that exposure to COVID-19 varies by occupation, gender, class and ethnicity. Three out of four workers in the most highly-exposed occupational groups, including health workers and care workers, are women, 6 of the 16 occupational groups are paid less than the national median hourly wage, and people from minority ethnic populations are “over-represented” in these highly exposed occupations (Office for National Statistics, 2020). However, this level of data disaggregation in national surveillance data systems is only seen infrequently.

There are few regularly reported national statistics on other health outcomes associated with the COVID-19 pandemic, either through its direct or indirect effects. For example, there is a widespread absence of surveillance data on sex-disaggregated rates of so-called ‘long-COVID’. Moreover, national surveillance systems are not regularly reporting other health impacts arising as a result of disruption to health systems and services, e.g. from lack of access to surgery, care for chronic conditions (cancer, diabetes, etc.), mental health impacts, or access to essential sexual and reproductive services including contraceptive and obstetric care.

This represents a major gap in our understanding of the health impacts, including the gendered impacts, of the pandemic. For example, recent research conducted in India to examine the impact of COVID-19 related lockdown on health care seeking behaviour shows that a significantly lower proportion of females visited hospitals during the lockdown period compared to the similar time of the previous year (Babu et al., 2020). Endler et al. (2019) reported evidence of a significant decline in access to a range of sexual and reproductive health services including contraceptive services and abortion services, and an increase in rights violations during the pandemic. Other studies (Kumari, Mehta and Chaoudhary, 2020; Bisht, Sarma and Saharia, 2020) also point towards the difficulties faced by pregnant women in seeking regular check-ups. However, apart from these individual studies, regular, comprehensive and nationally representative data on these other health consequences of the pandemic are mostly absent.

### Why are sex and gender important for health outcomes?

From the earliest days of the COVID-19 pandemic, national surveillance system data (WHO 2020b) and evidence from peer-reported studies (Chen et al., 2020; Jin et al., 2020)] noted a difference in outcomes in men and women. Compared to women of a similar age, men appeared to have a higher risk of severe infection and death. This was not entirely unexpected—disease outcomes are frequently unequal in men and women. In the previous coronavirus epidemics (e.g. SARS (Karlberg et al., 2004) and MERS (Chen et al., 2017)) similar differences were recorded. Such sex/gender differences are not limited to the coronaviruses but are seen in both communicable/infectious diseases and the non-communicable diseases (such as diabetes, heart disease, lung disease) (Mauvais-Jarvis et al., 2020)—with variously men or women experiencing the highest burdens and impacts depending on the nature of the exact pathogen or the disease itself.

Both sex and gender contribute to these differences in health status seen in people of all genders. Sex and gender interact across the lifecourse, and as Stefanick and Schiebinger (2020) point out, investigating the nature and extent of this interaction “enhances the quality of science, health, and medicine and contributes to global human health”. In the case of SARS-CoV-2 (the virus that causes COVID-19) analysis of sex/gender can provide important evidence on biological mechanisms influencing disease pathways in the human body (Takahashi et al., 2020), as well as identification of the social/structural drivers that influence risk and vulnerability (Adams, 2020; Stefanick & Schiebinger, 2020). It has been reported that immune system differences between males and females lead to differences in outcomes of COVID-19 infection (Scully et al, 2020) and may influence responses to vaccines (Gee et al, 2021). In part this may be due to women’s “heightened [biological] immune response compared to men”—something that may protect against severe disease and decrease risk of death (Mauvais-Jarvis et al., 2020), but could potentially also lead to more adverse vaccine reactions being suffered by women. Meanwhile gender, embedded in institutions and systems in society (Agarwal, 2021b), and embodied through behavioural norms and experienced at the level of the individual’s gender identity—what Connell (2015), refers to as the ‘gender order’ of any society—may drive differences *inter alia* in rates of exposure to the virus (and the power to isolate away from exposure), the likelihood of compliance with protective interventions (such as mask-wearing or vaccine uptake), patterns of care-seeking including testing uptake, and access to health services, including through characteristics such as service accessibility or affordability (Hawkes & Buse, 2020).

### Establishing a repository of sex-disaggregated COVID-19 data

In the earliest stages of the global COVID-19 pandemic, Global Health 50/50 (GH5050), an advocacy and accountability mechanism which collects evidence on gender-responsiveness and gender equality within the global health system, noted both the absence of sex-disaggregated data and no clear global system to systematically collect, collate and present such data. Global Health 50/50 established a reporting system in partnership with the International Center for Research on Women (ICRW, New Delhi, India) and the Africa Population and Health Research Centre (APHRC, Nairobi, Kenya) to report regularly on sex-disaggregated data at national level and in the case of India, Nepal and Afghanistan also at sub-national level (Global Health 50/50, 2020). The data tracker is now used regularly including by a number of international organisations as well as researchers and academics interested in sex, gender and COVID-19. For example, both UN Women and the United Nations Office for the Coordination of Humanitarian Affairs report the tracker’s data on their websites.

In the remainder of this paper we (authors from Global Health 50/50, ICRW and APHRC) report on the availability of data including in relation to the economic status of countries in the tracker, analyse sex-disaggregated findings along a pathway from prevention interventions (e.g. vaccines, testing) to infection outcomes (including hospitalisation and death), investigate the age- and sex-distribution of mortality, explore the relationship between gender and other social determinants in driving the observed male/female differences, and reflect on the limitations of what the data can (and cannot) reveal about the inequalities associated with the pandemic.

## Methods

### Data availability, collection and collation in the COVID-19 data tracker

Data are collected on a monthly basis from Government and national surveillance websites, online official reports, official media, including social media, and communications from Government sites. National sex-disaggregated data are collected on as many points as possible along the WHO-identified COVID-19 prevention-to-outcome pathway: namely, data on vaccinations, testing, cases, hospitalisations, admissions to intensive care, and recorded deaths. Additionally, we collect data disaggregated by age groups (where this is available) and infections in health workers. Each of these indicators is recommended by WHO for COVID-19 sex-disaggregated reporting by its member states (World Health Organization 2020a). Only cumulative data, as opposed to daily or weekly tallies, are collected. Data are entered into a standardised data entry tool. All data are reviewed by at least two people and undergo automated checks. Following verification, data are uploaded to our publicly available COVID-19 Sex-Disaggregated Data Tracker (Global Health 50/50, no date).

We currently report on data from over 200 countries. This includes the four countries of the United Kingdom (England, Scotland, Wales and Northern Ireland) and Hong Kong, all of which are listed as separate countries in the tracker as they have individual reporting mechanisms. Small countries, overseas territories and other countries reporting relatively few confirmed cases (< 500 total) are not currently covered by the tracker—except in the case of the Democratic People’s Republic of Korea, which reports no cases but is still monitored regularly for data. Countries are included irrespective of whether they sex-disaggregate their data—but the extent of sex-disaggregated data (or its absence) is noted for each country and each variable (i.e. vaccination, testing, cases, hospitalisation, death). Data beyond the binary sex categories of male/female is rare. We have reported a ‘non-binary’ category for vaccine uptake in Austria, and the States of Tamil Nadu and Haryana in India provide surveillance data on COVID-19 in transgender people. In the United States of America case and death surveillance data includes the category ‘other’ but this is not further defined (CDC, 2021a, b).

The 200 countries included in the Tracker account for 99.9% of all reported COVID-19 cases globally and 99.9% of recorded deaths (Worldometer, 2021). By August 2021 we located sex-disaggregated data for 67% of global cases and 81% of global deaths. A small number of countries report sex-disaggregated data at least once on: testing (8%; 16/200); hospitalisations (14%; 28/200); intensive care admissions (10%; 19/200), health care workers (6%; 11/200 and vaccine uptake (22%; 43/200). Countries report sex-disaggregated data inconsistently and incompletely across all key indicators. Only six countries (Estonia, Israel, New Zealand, Northern Ireland, France, Denmark) have ever reported sex-disaggregated data for all key indicators on the testing-to-outcome pathway—and none have done so consistently for an extended period of time.

**Fig. 1:**
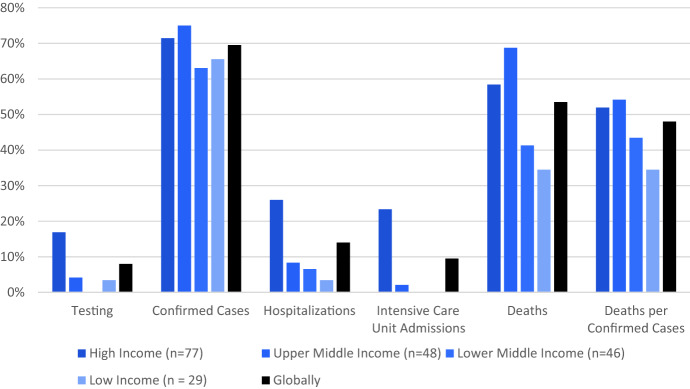
Percent of Countries Tracked Reporting Key COVID-19 Clinical Pathway Indicators categorised by World Bank Income Grouping

Analysis of the availability of sex-disaggregated data according to World Bank income status of the reporting country, shows that for cases and deaths, low-income countries have the lowest level of data availability. For testing, hospitalization and ICU admissions, high-income countries have the highest level of data availability—see Fig. 1.

### Age-and sex-distribution

Fifty countries have reported death data by age and sex since May 2021. However, countries do not provide consistent age groupings. Instead of looking at the overall age and sex data for deaths, we were compelled to form two groups of countries; Group 1 (37 countries) including ages 20–49 years and age 50 years and over, and Group 2 (17) ages 15–44 years and age 45 years and over. These groupings are not mutually exclusive as six countries provided adequately fine age groupings to allow inclusion in both groups (England, Mexico, Nigeria, Philippines, USA and Ukraine).^1^ For all countries, deaths among children (either less than 15 years or less than 10 years) were rare and were excluded from analysis.

### Relationship between gender inequality, other structural drivers and the COVID-19 mortality gap

We analysed the relationship between a selection of structural determinants and the COVID-19 mortality ratio between males and females (i.e. ratio of COVID-19-related male deaths:female deaths)—these determinants included the country’s income classification (World Bank grouped), the gender inequality status of the country, proportion of women in the paid workforce (see work of Adams, 2020) and proportion of the country that is urbanised.

The Gender Inequality Index (GII) from the United Nations Development Program (UNDP, not dated) provides a measure of the level of gender inequality in a country by assessing three areas: reproductive health, political and social empowerment and economic status (female participation in the paid workforce). Measured from 0 to 1, the closer the value to 0 the more gender equal a country. For country income level classification, our analysis uses the 2020–2021 World Bank income groupings (World Bank, 2021)—low-, lower-middle, upper-middle, and high-income. We report on the availability of data by country income level, as well as COVID-19 outcomes by country income level.

Recognising that any observed differences in COVID-19 mortality may simply reflect and replicate existing life expectancy differences between men and women in any country, we analysed the pre-COVID life expectancy ratio (i.e. ratio of male:female life expectancy) in relation to the same structural determinants (gender inequality, female labour force participation, country income level).

Full details of methods and data sources are presented in Appendix 1.

### Methods of statistical analysis

Descriptive analyses to summarize gender differences seen across key indicators consisted of constructing population-adjusted proportions and gender ratios. Tabulations were used to compare performance in data availability and gender disparity across indicators between World Bank income groupings. Pearson correlations were computed to assess the relationship between the gender disparities in COVID-19 mortality and country income level, GII, female labor force participation and the percent of population living in an urban area. All analysis was conducted in Stata13 (StataCorp; College Station, TX).

## Limitations to the data

There are a number of limitations to the accuracy of the data. The total number of cases and deaths reported in the tracker may differ from the most recent numbers reported by a country for two primary reasons. Firstly, we record the most recent data where sex-disaggregation is provided – which may not be consistent with more regular, cumulative, updates from countries. Secondly, these figures reflect only the total number of cases/deaths where sex-disaggregated data is available, which may only be a portion of total numbers in some countries. Reporting of data is also limited to what countries themselves make available, which, as noted, is categorised as male/female (i.e. no mention of transgender and/ non-binary categories).

A major limitation is the lack of data that would allow a more in-depth intersectional analysis of the data and an absence of data on secondary health impacts. In the tracker we have found very few examples of country data that takes a more intersectional lens to the surveillance system (Kapilashrami and Hankisvsky, 2018). While 55 countries do report both age and sex, no countries report specifically on rural/urban distribution. Migration status is not recorded in COVID-19 surveillance data, although some countries (including those with high numbers of migrants) do report whether or not any individuals are considered to be nationals or “other” (including visitors, migrants, etc.). Finally, countries do not provide in-depth information about occupation and COVID-19 which might, for example, allow for investigation of the relationships between the gendered hierarchies of the health professions and risks of COVID-19.

In the case of other impacts, data are also absent. For example, we do not find regularly reported data that records pregnancy status and COVID-19 outcomes. In the case of the HIV epidemic, sentinel surveillance systems that capture data from antenatal clinics are an important source of understanding the impact of HIV on women of reproductive age, including the impact on their reproductive histories, as well as identifying the overall spread of the epidemic in the population (Thamattoor et al, 2015). For COVID-19, however, such sentinel surveillance systems are not in place, and the impact on pregnant or breastfeeding women and their infants is captured through cross-sectional surveys only (and through national data in the case of the USA and The Philippines^2^).

## Results

### Sex-distribution along the testing-to-outcome COVID-19 pathway globally

**Fig. 2:**
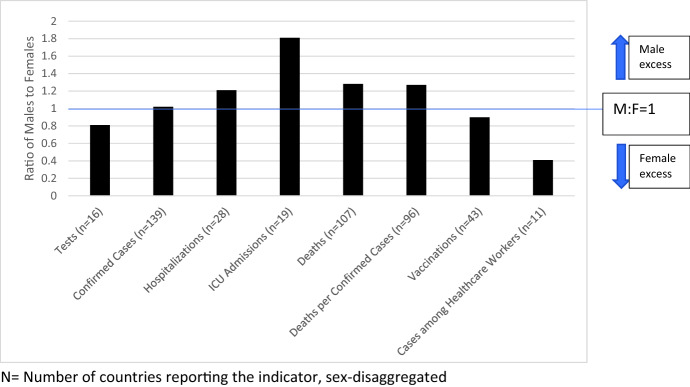
Global Male to Female Ratios for Key COVID-19 Indicators

Women are more likely to be tested (male: female ratio = 0.81:1, *n* = 16 countries), but account for slightly fewer cases (M:F = 1.02:1, *n* = 139 countries). Men are more likely to be hospitalised (M:F = 1.21:1; *n* = 28 countries), more likely to be admitted to intensive care is more than double that of women’s (M:F = 1.91:1; *n* = 19 countries), and more likely to die (M:F = 1.28:1; *n* = 107 countries). To date, more women have received vaccinations (M:F = 0.90:1; *n* = 43 countries). Infections in healthcare workers are skewed heavily towards women being more likely to be infected: 4 male healthcare workers becoming infected for every 10 female healthcare workers (*n* = 11 countries) (Fig. 2).

The M:F ratio of deaths is 2.08:1 in low-income countries, falling to 1.21:1 in high-income countries. A similar pattern is seen for diagnosed cases too, although the difference between rich and poor countries is less pronounced: the M:F ratio of cases in low-income countries is 1.41:1, falling to 0.92:1 in high-income countries—see Table 1.

**Table 1:** Sex-adjusted sex ratios (M:F) in confirmed cases and deaths, by income level as of July 2021 (*n* = number of countries)

	Confirmed cases	Deaths
World bank income level		
High income (*n* = 77)	0.92	1.21
Upper middle income (*n* = 48)	0.98	1.36
Lower middle income (*n* = 46)	1.39	1.47
Low income (*n* = 29)	1.41	2.08

**Fig. 3:**
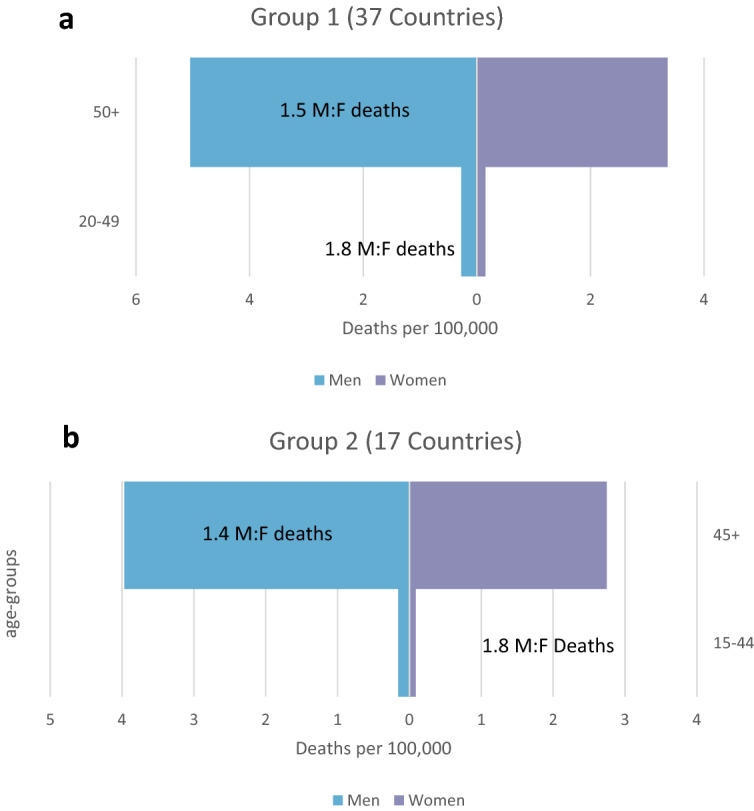
**a** (Group 1 countries) and **b** (Group 2 countries). Male: Female mortality ratio per 100,000 population by age and sex in Group 1 and Group 2 countries

### Age- and sex-disaggregated data combined

Fifty countries have reported updated data disaggregated by both age and sex since May 2021. Data show that adult men have higher COVID-19 mortality rates than adult women across all adult ages though the gap between men and women declines with age. Figure 3a, b shows deaths per 100,000 by age and sex for the two groups of countries based on available age and sex data for deaths. In 34 of the 50 countries men have higher death rates at all ages (including across the reproductive ages). In 13 countries women have higher mortality rates in only one or two age groups—generally in the age range 10–30 years old. In five countries (Bangladesh, Jordan, South Africa, Ukraine and Vietnam) women had higher mortality rates compared to men in almost all reproductive age groups, although men had higher rates in the older age groups. For three countries the numbers of deaths outside of the oldest age groups were too small to undertake comparative analysis.

### Sex-distribution of cases in health workers

**Fig. 4:**
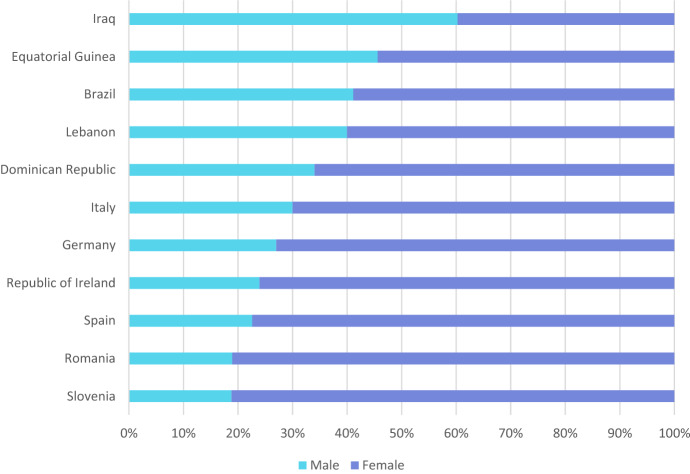
Sex-disaggregated distribution of COVID-19 cases in health workers, 11 countries

In 10 of the 11 countries reporting infections in health workers, women form the majority of cases in this occupational category—see Fig. 4.

### Structural drivers of the COVID-19 mortality gap and the pre-COVID life expectancy gap

Data on the COVID-19 mortality ratio recorded in the last 3 months was available for 97 countries; and pre-COVID life expectancy data are available for nearly all countries (*n* = 185). Using the UNDP Gender Inequality Index (GII), we found a significant, positive relationship between the COVID-19 M:F mortality ratio and GII. The higher values of the GII, indicating higher levels of gender inequality at country level, are significantly associated with a higher ratio of male to female deaths.

We also found a significant correlation between the COVID-19 M:F mortality ratio and proportion of the population urbanised (urbanisation is associated with lower mortality ratios). There is no significant association with female labour force participation.

The pre-COVID life expectancy ratio is not significantly correlated with any of the variables gender inequality, country income level, proportion labour force participation or proportion urbanisation—see Table 2.

**Table 2:** Correlation coefficients—male:female mortality ratios and structural determinants

	COVID mortality ratio	Pre-COVID life expectancy ratio	Pre COVID life expectancy ratio
	*n* = 97	*n* = 97	*n* = 185
Gender inequality index	0.41**	− 0.004	0.03 (*n* = 162)
Income level	− 0.38**	0.02	0.003 (*n* = 185)
Female labor force participation	0.03	− 0.08	0.01 (*n* = 177)
Percent of population living in urban area	− 0.49**	0.07	0.01 (*n* = 183)

*n* = no of countries included in analysis

^**^Denotes *p* < 0.001

Comprehensive sex-disaggregated data combined with gender analysis can illustrate sex and gender-associated health inequities and can be used for examination of possible reasons behind the observed differences. For example, data in the surveillance tracker have shown that in the small number of countries reporting sex-disaggregated testing data, women are slightly more likely to be tested compared to men. This is congruent with other studies showing gendered patterns of healthcare-seeking and health protection: men generally have a lower use of health services, including for prevention and screening interventions, compared to women (Teo et al., 2016). Reasons for men’s apparent under-utilisation of testing services deserves further investigation, but highlights the need to have more gender-responsive public health communications and interventions. Identifying and addressing the social and structural barriers to low rates of access to testing services (e.g. such services are only open during formal working hours) has been shown to be more effective than simply increasing communications telling people to ‘go get tested’. For example, to increase HIV testing among men in Tanzania, Conserve and colleagues (2019) report a range of changes that the health system could make to increase men’s accessibility to services.

The tracker data globally have revealed men’s higher rates of hospitalisation and admission to intensive care units. In part this may reflect underlying biological vulnerabilities: men’s immunological responses to viral infections are known to be less strong compared to women’s responses (Klein & Flanagan, 2016). But in addition to sex, gender may also be playing a role—including in providing explanations for women’s possible under-representation in the data. For example, the lower rates of hospitalisation in women compared to men may reflect women’s lack of power within families and across society resulting in unequal access to hospital services and to intensive (and costly) interventions within the health system. Such findings have previously been noted in the case of health-care pathways across a range of conditions (Saikia, Moradhvaj and Bora 2016), and the sex-disaggregated results from the data tracker deserve further investigation and analysis of this potential source of bias and inequality.

Moving beyond sex-disaggregated data, in the 55 countries where data are disaggregated by both age and sex we can examine patterns in mortality risk. For the majority of the 55 countries men are at higher risk of death at all ages, but in a smaller number of countries there is evidence that women in the reproductive age groups have higher mortality rates (although this is reversed again in the older age groups). These findings do not currently provide any robust evidence of the potential adverse impact of COVID-19 among women of reproductive age, including on their pregnancy outcomes—as reported in the studies from cross-sectional surveys—but highlight both the importance of further investigation as well as the need to have more rigorous and comprehensive data disaggregated by age, sex and ideally by pregnancy status.

Our analysis of the relationship between gender equality status of a country and male:female COVID-19 mortality ratios (Fig. 4) finds that the closer the country is to gender equality (as measured within the Gender Inequality Index of UNDP), the smaller the mortality gap between men and women. In highly gender unequal countries, the mortality gap is greater. This finding may not be as unexpected as it first appears. Although we did not see a relationship between GII and pre-COVID mortality ratios, other authors have noted statistically robust relationships between gender equality status and health behaviours (both harmful and protective) and health outcomes. For example, at aggregate national levels there is evidence of a relationship between gender equality/inequality and tobacco-smoking ratios (i.e. the ratio of female:male tobacco smokers in a population). Hitchman and Fong (2011) examined cross-national associations between the United Nations Development Programme’s gender equality measure (GEM) and female:male smoking prevalence ratios. Adjusted for levels of economic development and income inequality, their models suggested that gender equality at national level is associated with a smaller female:male smoking prevalence gap—in other words, women forming a higher proportion of all smokers the closer the country moves to gender equality.

Evidence from the earliest studies in the pandemic till now have shown an association between the presence of existing non-communicable disease (such as heart disease, lung disease and some metabolic diseases) and a higher risk of death from COVID-19 (Clark et al, 2020)—which may go some way to explaining men’s higher mortality rates compared to women’s rates. Applying a gender lens to understanding the excess mortality in men, the role of gendered lifetime risk exposure to tobacco, alcohol, air pollution, and other environmental and behavioural risks requires further analysis. Analysis over several decades has shown that the non-communicable diseases are driven by the intersection of poverty, inequality and education, as well as gender (Barbeau 2004; Cortese & Ling, 2011; Williams et al., 2018). But at a structural level these exposures are also driven by commercial interests seeking to derive a profit from the sale of unhealthy products such as tobacco, alcohol and ultra-processed foods—exposures that may be more often experienced by men in many settings (Allen et al., 2017).

Adams (2020) in her cross-national analysis of the relationship between gender equality and COVID-19 mortality in OECD countries found that ““the percent of the full-time workforce comprised by women is positively related to the percent of female Covid-19 deaths across countries”. Our own analysis did not find a statistically significant association between the COVID-19 mortality ratio and women’s labour force participation. We did, however, find that the mortality ratio was associated with the country income level and proportion of the country urbanised. The mortality ratio declines as countries become richer and more people live in urban areas—both variables which may, of course, be associated with increasing gender equality at the aggregate national level. Chant (2016) characterises (with reservations) cities as ‘good for women’ and highlights the widespread understanding that ‘urbanisation is conducive to greater gender equality’ (p.21).

An alternative hypothesis worth exploring is that the poor functioning of vital registration systems may be disproportionately impacting the likelihood that women’s deaths are being recorded (Jackson et al., 2018). Interventions to improve women’s access to vital registration systems, including mobile registration services staffed by women in Pakistan (International Foundation for Electoral Systems 2013), conditional cash payments which have increased the registration of girl children (Baruah et al., 2013) represent the type of gender-responsive solutions that require consideration when addressing the chronic problem of women’s access and presence within the types of vital registration systems on which COVID-19 and all kinds of mortality data rely.

In summary, our analysis of the relationships between structural determinants of health (including economic, social and occupational determinants) and the male:female mortality gap in the COVID-19 pandemic has identified areas that warrant further in-depth analysis—including analyses conducted at national or sub-national level, and further examination of interactions and confounders.

Our analyses of the role that gender plays, including through its intersection with other structural determinants, are only possible because we have a foundation of sex-disaggregated data on which to build hypotheses and proposals for additional investigation. The International Health Regulations (IHR), adopted in 2005, oblige all WHO Member States to meet basic minimum surveillance requirements—defined as “the systematic ongoing collection, collation and analysis of data for public health purposes and the timely dissemination of public health information for assessment and public health response as necessary” (WHO, 2005). The regulations do not, however, stipulate that surveillance data should be sex-disaggregated. Recognising the importance of sex and gender to the COVID-19 pandemic, the WHO COVID-19 monitoring and evaluation framework published in June 2020 and aiming to provide guidance to countries on systems indicators to “monitor preparedness, response and situations” recommends collection and reporting of sex-disaggregated data on testing, cases, deaths, hospitalisation, case fatality rates, and case rates in health workers (WHO 2020a). Our review of national surveillance data has highlighted the lack of compliance with this WHO recommendation.

## Conclusion

Sex-disaggregated data combined with gender analysis can help to monitor one aspect of the distribution of, and vulnerability to, the COVID-19 pandemic, as well as identify whether or not interventions are working equitably. At a minimum, sex-disaggregation of public health data is a core component of gender-responsive accountability mechanisms. By applying a more intersectional lens to addressing health generally and COVID-19 in particular our understanding of and response to this pandemic will be more equitable and effective. Despite the multiple benefits of sex-disaggregated data, however, our review of national surveillance system outputs has highlighted the relative absence of such fundamental data in many health systems. Unlike more intersectional data on other social and structural determinants of health and wellbeing, such as ethnicity, wealth, occupation or education, data on sex or gender (or sometimes both) are a core existing variable for much of clinical and public health practice. In our collective experience (across many economic regions), health system data—including data on hospital admissions and death certificates—at a minimum includes data on sex (and usually on age too). This puzzling absence of sex-disaggregation in an age of data surveillance, data harvesting and analysis on an unprecedented scale, likely reflects more on the low priority accorded to understanding and addressing sex and gender in health systems, rather than a failure to collect such data in the first place. It may also, of course, also reflect the inherent and long-standing biases within the health and medical systems and professions whereby the ‘norm’ of the male body has been the default for millennia and there has been a well-described lack of attention paid to issues of both sex and gender.

COVID-19 can serve as wake-up call to the health sector to overcome its longstanding gender-blindness and ensure routine reporting of sex-disaggregated data. This will inform both policy and practice and is in line with WHO recommendations on sex-disaggregated reporting on COVID-19. Moreover, such data is necessary to make good on the SDG pledge to hold governments to account and leave no one behind.

We recognise, however, that reporting, analysing and acting on sex-disaggregated data is not cost-free. As a global pandemic, COVID-19 further reinforces the fact that sex-disaggregated data are a global public good and as such may require international collaboration and support to some regions and countries to develop/strengthen systems to report sex- and age-disaggregated data. Our tracker has shown that the low-income countries in general are lagging behind other regions, and may require additional (and immediate) capacity strengthening and resource allocation.

Presently the international community lacks a global architecture to facilitate the rapid and efficient sharing of data from countries. We make two recommendations to overcome this weakness. First, while the International Health Regulations (IHR) stipulates the legal obligations of States to inform WHO about the occurrence of certain public health events, there is currently no reporting mechanism that enables data exchange from public health institutes and agencies directly to WHO. We therefore encourage the independent expert committee established by WHO (UN News, 2020) to review the IHR, to on the one hand reinforce the call on countries to report sex disaggregated data and, on the other hand, to use the review as an opportunity to enshrine within the IHR a system, administered by WHO, to collate and centrally publish country-reported data. Second, while Global Health 50/50 have been able to fill in some of the sex-disaggregated COVID-19 data gaps over the past year, the pandemic reveals that a more formal and sustainable system for reporting sex-disaggregated data is long overdue—and not just for outbreak data. While this will require additional resources, if the global health community is committed to gender equality, then supporting sex-disaggregated data should be a central part of effective, evidence-informed and equity-focused responses to this and other urgent health problems.

## Data Availability

All data are available on the COVID-19 sex-disaggregated data tracker accessible at: https://globalhealth5050.org/the-sex-gender-and-covid-19-project/

## Footnotes

1 Group 1: Afghanistan, Australia, Bosnia and Herzegovina, Cambodia, Cabo Verde, Canada, Colombia, Cuba, Czech Republic, Denmark, Ecuador, England, France, Guatemala, Haiti, Hong Kong, Hungary, Israel, Italy, Kenya, Lithuania, Maldives, Mexico, Moldova, Nigeria, Peru, Philippines, Portugal, Slovakia, South Africa, Spain, Switzerland, USA, Uganda, Ukraine, Vietnam and Wales. Group 2: Austria, Bangladesh, Cabo Verde, Chad, Ecuador, England, Equatorial Guinea, Guinea-Bissau, Jordan, Mexico, Nepal, Nigeria, Philippines, Scotland, Slovenia, USA and Ukraine.

2 The national surveillance data does not allow consistent analysis of the impact of COVID-19 on pregnancy status. In two countries, the United States of America and the Philippines, we are able to compute pregnancy-related morbidity and mortality outcomes. The USA reports 1.17 COVID-19 deaths for 1000 cases among pregnant women as of 9 Aug 2021 (CDC, 2021). While the data from the USA does not provide the ability to look at deaths per 1000 cases among women age 15–49, we can compute deaths per 1000 cases for women age 10–49 years, 0.98, or women aged 20–49 years. In comparison, The Philippines reports 3 deaths among 109 cases among pregnant women (17 August 2021), which gives a rate of 27.5 deaths per 1000 cases among pregnant women compared to 3.5 deaths per 1000 cases women age 15–49 years in the Philippines (NCVO tracker). The somewhat disparate findings in the two countries underscores the need for consistent data on outcomes among pregnant women globally.

## Code availability

All data are accessible through an API at: https://api.globalhealth5050.org/docs/
